# Use of Thiols in the Treatment of COVID-19: Current Evidence

**DOI:** 10.1007/s00408-021-00465-3

**Published:** 2021-08-27

**Authors:** Mario Cazzola, Paola Rogliani, Sundeep Santosh Salvi, Josuel Ora, Maria Gabriella Matera

**Affiliations:** 1 Unit of Respiratory Medicine, Department of Experimental Medicine, University of Rome “Tor Vergata, Rome, Italy; 2 Department of Clinical Research, Pulmocare Research and Education (PURE) Foundation, Pune, India; 3 Unit of Pharmacology, Department of Experimental Medicine, University of Campania “Luigi Vanvitelli, Naples, Italy

**Keywords:** COVID-19, Erdosteine, N-acetylcysteine, Oxidative stress

## Abstract

There is a possible role for oxidative stress, a state characterized by an altered balance between the production of free radicals or reactive oxygen species (ROS) and antioxidant defences, in coronavirus disease 2019 (COVID-19), the genesis of which is quite complex. Excessive oxidative stress could be responsible for the alveolar damage, thrombosis, and red blood cell dysregulation observed in COVID-19. Apparently, deficiency of glutathione (GSH), a low-molecular-weight thiol that is the most important non-enzymatic antioxidant molecule and has the potential to keep the cytokine storm in check, is a plausible explanation for the severe manifestations and death in COVID-19 patients. Thiol drugs, which are considered mucolytic, also possess potent antioxidant and anti-inflammatory properties. They exhibit antibacterial activity against a variety of medically important bacteria and may be an effective strategy against influenza virus infection. The importance of oxidative stress during COVID-19 and the various pharmacological characteristics of thiol-based drugs suggest a possible role of thiols in the treatment of COVID-19. Oral and intravenous GSH, as well as GSH precursors such as N-acetylcysteine (NAC), or drugs containing the thiol moiety (erdosteine) may represent a novel therapeutic approach to block NF-kB and address the cytokine storm syndrome and respiratory distress observed in COVID-19 pneumonia patients

## Oxidative Stress and COVID-19

Oxidative stress is a state characterized by an altered balance between the production of free radicals or reactive oxygen species (ROS) and antioxidant defences that can also cause tissue damage when natural defences are defeated by external insult [1].

Indeed, cells are protected against oxidative stress by a sophisticated enzymatic and non-enzymatic antioxidant defence system that counteract and regulate overall ROS levels to maintain physiological homeostasis [2].

Glutathione (GSH), a low-molecular-weight thiol that is present in cells at millimolar concentrations, is the most important non-enzymatic antioxidant molecule [3]. As a carrier of an active thiol group, GSH is able of inducing detoxification of ROS, reactive nitrogen species (RNS) and other free radicals. It has been called the “master antioxidant” because it maintains a reduced environment in the lungs despite being constantly exposed to high levels of environmental oxygen. GSH is involved in modulating the release of proinflammatory cytokines by regulating several transcription factors. It also enhances the activity of immune cells and functions as an antioxidant within them. Furthermore, GSH plays a vital role in the repair of damaged DNA by replacing the missing electron.

There is much discussion about a possible role for oxidative stress in coronavirus disease 2019 (COVID-19) [4]. In hospitalized patients with COVID-19, a significant association between oxidative stress biomarkers and disease severity has been demonstrated [5]. Elevated levels of oxidative stress and reduction of antioxidant indices can aggravate COVID-19 severity [5]. A small study involving nine critically ill COVID-19 patients showed that the systemic oxidative stress state is strongly altered, with an increase in lipid peroxidation, but also a deficit in some antioxidants (vitamin C, GSH, thiol proteins) and trace elements (selenium) [6].

The genesis of oxidative stress in patients with COVID-19 is quite complex. It is known that SARS-CoV-2 attaches itself to human epithelial cells through non-covalent binding of its spike protein to the receptor of angiotensin-converting enzyme 2 (ACE2) on the host cell [7]. Then, due to viral evasion of the interferon (IFN)-I/III response, prolonged and extensive replication of SARS-CoV-2 occur in lung epithelial cells and vessel endothelial cells [8].

After attachment and virion membrane fusion, ACE2 expression is downregulated and there is a local or systemic depletion of the enzyme known to cleave the peptide hormone angiotensin-II (ATII). As a result, ATII levels increase because ATII without the compensatory action of ACE2 is not converted to angiotensin1-7 [9]. ATII overactivates its Ang II type 1 receptor (AT1R) promoting vasoconstriction and a sudden acute increase in circulating levels of different proinflammatory cytokines including IL-6, IL-1, TNF-α, and the “cytokine storm” [10].

The “cytokine storm”, in turn, induces massive recruitment of neutrophils and cells from the mononuclear phagocyte system (MPS), such as macrophages, monocytes, and immature dendritic cells, into the inflamed tissue [7]. IL-6, proinflammatory cytokines, NO and several chemokines activate vascular endothelial cells, platelets, and neutrophils, ultimately forming platelet–neutrophil complexes [11]. The interplay between vascular endothelial cells, activated platelets and activated primed neutrophils produces a highly coagulative and inflammatory state described as immunothrombosis. The migration of neutrophils and platelets into the pulmonary microvasculature and into lung tissues results in severe damage to the epithelial layer, alveolar fibrin deposition and the formation of microthrombi [11].

Activated neutrophils and MPS cells produce large amounts of ROS, through nicotinamide adenine dinucleotide phosphate (NADPH) oxidase, which is the major source and primary trigger for ROS generation, thereby creating an imbalanced oxidative stress response [9]. Excessive oxidative stress might be responsible for the alveolar damage, thrombosis, and red blood cell (RBC) dysregulation seen in COVID-19 [12].

In effect, free radicals, the downstream product of cytokine storm, are the molecules that are ultimately responsible for damage to cells and various organs [13]. In addition, the overproduction of ROS suppresses the T lymphocyte response, which results in impaired adaptive immunity [14]. Furthermore, during viral replication, the mechanisms of genome expression disturbed by the high rate of free radicals activated by the oxidative stress would act on the proteins resulting from the translation of messenger RNAs and would completely change the structure of the SARS-CoV-2. Oxidative damage could thus induce viral mutations that could minimize the effect of the immune system [14]. Such mutations may involve the non-structural proteins or structural proteins such as the spike protein, which favours the evolution of the COVID-19 pandemic.

In low-risk individuals, an excess of ROS is counterbalanced by an increase in antioxidant defences [12]. GSH can prevent damage to important cellular components caused by ROS and their derivatives [15]. Its anti-inflammatory effects are exerted through the inhibition of ACE activity, decrease of ROS production and reduction of nuclear factor-kB (NF-kB) activation. Consequently, GSH has the potential to keep the cytokine storm under control [15].

However, in subjects with impaired redox balance, ROS production is not properly controlled, leading to RBC membrane peroxidation, which in turn perpetuates neutrophil activation [12]. It has been suggested that GSH deficiency is a plausible explanation for serious manifestations and death in COVID-19 patients [16]. Age, comorbidities, smoking, and dietary factors are the most common causes responsible for endogenous GSH deficiency and the mechanisms through which this deficiency may contribute to the pathogenesis of severe COVID-19 disease [16]. Furthermore, men, who are significantly more likely to suffer severe effects of COVID-19 infection and experience a higher mortality rate than women [17], have lower plasma levels of reduced GSH than women [16].

Intriguingly, it has been suggested that also many psychological, environmental, and physical factors causing stress may worsen the effects of COVID-19 by inducing the generation of oxidative stress [14]. Chronic stress could stimulate the conserved transcriptional response to adversity through the sympathetic nervous system. This leads to the induction of proinflammatory cytokines that cause inflammation and ROS generation, thereby producing an imbalanced oxidative stress response and, ultimately, a cytokine storm.

However, the documentation generated by a computational study that a pro-oxidant environment with low GSH levels would promote cellular entry of viruses is certainly more intricate for its possible therapeutic implications. Indeed, the binding of the SARS-CoV/CoV-2 spike protein to ACE2 is maximal when the sulphur groups of ACE2 are in the form of disulphides and significantly impaired when it is completely reduced to sulphhydryl (SH) groups [18]. It has been hypothesized that the age-dependent decline of low-molecular-weight thiols, such as GSH and its biosynthetically related compounds [cysteine (Cys), γ-glutamylcysteine, cysteinylglycine], in extracellular fluids would not only be the actual causative event but also a molecular marker of increased risk of infection and development of serious COVID-19 [19].

## Properties of Thiols

When extracellular and/or intracellular levels of GSH are abnormal, one therapeutic approach is to administer GSH orally. This approach induces increases in GSH concentrations that occur very rapidly but return to basal levels within 4 h. Such a short half-life does not lend itself well to treatment that must be continued for quite a long time [3]. Furthermore, the pulmonary availability of oral GSH is affected by the expression of the transporter that takes up GSH and transports it into the ELF [3].

One possible alternative is to stimulate endogenous GSH synthesis. It occurs through a two-step enzymatic process that requires ATP [20]. In the first step, Cys is conjugated to glutamate, generating γ-glutamylcysteine. In the second step, glycine is added to γ-glutamylcysteine to form GSH.

Unfortunately, Cys undergoes rapid oxidation in solution, generating the inactive disulphide, cystine (Cys-Cys) [21]. However, drugs containing the thiol moiety (-SH), such as N-acetylcysteine (NAC) and erdosteine, can also act as antioxidant drugs directly through free –SH groups that serve as a source of reducing equivalents, as well as indirectly through the replenishment of intracellular GSH levels [22].

It has been noted that the acetylation of the N-terminal of Cys, which is the synthesis of NAC, confers sufficient stability to the molecule to facilitate delivery of reduced SH moieties. There are two advantages of NAC over Cys in GSH synthesis: the SH group of NAC remains reduced more than the SH group of Cys and the transport of NAC through cell membranes occurs much more easily [21]. However, when using NAC to increase endogenous GSH synthesis, one must always keep in mind that only a small fraction of NAC can permeate the membrane prior to hydrolysis to Cys in the intracellular environment, and that, in any case, the rate-limiting step in GSH synthesis involves the conjugation of Cys to l-glutamate [21]. Thus, increasing the dose of NAC does not necessarily lead to an increase in GSH synthesis.

Erdosteine contains two S atoms, one of which is a thioether in the aliphatic side chain and the other is enclosed in the heterocyclic ring (thiolactone) [23]. The thiol group is present in a lactone ring, which after being metabolized in the liver becomes available for free radical scavenging and antioxidant activity.

Accumulating evidence suggests that NAC [24] and erdosteine [23], which are also classified as mucolytic agents, being thiol drugs, exhibit multiple pharmacological actions of relevance to the treatment of a range of respiratory diseases including COPD, asthma, and IPF. In addition to potent antioxidant and anti-inflammatory properties, such drugs also elicit antibacterial and antiviral activity and are also able to influence bronchial tone [22].

Thiols may modulate inflammation and oxidative stress by acting through the NF-kB/inhibitor of the NF-kB (IkB) pathway [25]. It has been suggested that they inhibit oxidative stress by acting as a direct scavenger of ROS and changing the cellular redox state. This, in turn, may influence NF-κB activation and modulate the inflammatory response [26].

When investigating the pharmacological properties of NAC that impact oxidative stress and inflammation using isolated human bronchi, it was observed that NAC administered at medium-to-high concentrations was effective in preventing the reduced antioxidant response induced by lipopolysaccharide (LPS) in an in vitro model of COPD exacerbation, increasing GSH levels by ≈50%, and improving superoxide dismutase activity by ≈150% and total antioxidant capacity by 90% compared with bronchi incubated with LPS alone [27]. Medium–high concentrations of NAC also significantly reduced the pro-oxidant response induced by LPS by decreasing the levels of peroxidase activity by ≈30% and the levels of H_2_O_2_, malondialdehyde (MDA), and nitric oxide (NO) compared with bronchi incubated with LPS alone. NAC administered at high concentrations significantly inhibited the release of interleukin (IL)-1β, IL-8, and tumour necrosis factor-α (TNF-α) induced by LPS incubation. At lower concentrations NAC significantly reduced the release of IL-6 induced by stimulation with LPS.

Using the same experimental model, it was shown that challenging the airways with LPS induces ROS formation and neurogenic inflammation leading to the release of neurokinin A (NKA) [28]. NAC at concentrations ≥ 5 μM (corresponding to the plasma level after oral administration of NAC 200 mg/day) significantly inhibited the levels of NKA. The effect of low concentrations of NAC on the NKA levels was significantly associated with increased concentrations of GSH, whereas only NAC administered at 35 μM, corresponding to the plasma level after oral administration of NAC 1200 mg/day, normalized the peroxidase activity and the levels of H_2_O_2_, MDA, NO, total antioxidant capacity, and IL-6 after an overnight challenge with LPS. However, NAC, and the relative modulation of NKA, had no significant effect on superoxide dismutase activity. Interestingly, NAC elicited specific anti-inflammatory activity against IL-6 yet at low concentrations, corresponding to the oral administration of NAC at 200 mg/day.

Erdosteine has also been shown to inhibit LPS-induced TNF-α, IL-1β, IL6, and free radical production in rat alveolar macrophages [29] and attenuate the increase of inflammatory cells in the BALF of rats following instillation of LPS [30]. Another study showed the ability of erdosteine to inhibit LPS-induced apoptosis of rat bronchial epithelium [31]. The effect of erdosteine was stronger than that of NAC.

These results suggest that thiols possess relevant antioxidant and anti-inflammatory properties that are worth considering in the treatment of patients with COVID-19. Furthermore, they also show antibacterial and antiviral activity.

It is now clear that some bacteria have evolved not only to survive the oxidative stress encountered during infection but also in some cases to use it to thrive during infection [32]. It is, therefore, of interest that NAC and GSH dramatically increase the killing of *Staphylococcus aureus* by neutrophils in vitro [33]. This effect is not necessarily due solely to protection of cells from self-induced oxidative stress. One possible explanation is that NAC and GSH reduce the concentration of extracellular NO. Furthermore, NAC plays a role in the various steps of biofilm formation, such as adhesion to inert and living surfaces, matrix production and organization, and dispersal of preformed biofilms, and reduces the production of extracellular polysaccharides, the major structural components of the biofilm of most bacteria [34].

Erdosteine also exerts antibacterial effects by affecting the integrity of pilin molecules by the interaction between the -SH group and the intrachain disulphide bonds of pilins. This can induce a morphological change in pilin structure that interferes with the binding of bacterial fimbriae to the receptor [23]. In addition, erdosteine significantly reduces both *S. aureus* and *Escherichia coli* adhesiveness to human mucosal epithelial cells via inhibition at the fimbrial level [35].

Thiol-based drugs may also be an effective strategy against influenza virus infection because virus-induced oxidative stress is important in the regulation of the host immune system and in the pathogenesis of pulmonary damage during influenza virus infections [36]. There are no data on the ability of erdosteine to prevent viral episodes, but an old trial showed that administration of NAC during the winter provides a significant attenuation of influenza and influenza-like episodes, especially in elderly high-risk individuals [37]. NAC did not prevent A/H1N1 virus influenza infection but significantly reduced the incidence of clinically apparent disease and increased cell-mediated immunity.

It has been documented that NAC inhibited replication of influenza (strains A and B) and respiratory syncytial virus and, in a dose-dependent manner, the induction and the release of MUC5AC, IL-8, IL-6, and TNF-α after virus infection [38]. It also decreased the intracellular H_2_O_2_ concentration and restored the intracellular total thiol contents. Mechanisms of NAC included inhibition of NF-κB translocation to the cellular nucleus and phosphorylation of p38 MAPK. However, according to the Oxford COVID-19 Evidence Service Team, the therapeutic efficacy of NAC is likely to be strain dependent [39]. As there is little evidence on the efficacy of NAC as an antioxidant in influenza and other acute viral infections of the respiratory tract it is difficult to draw solid conclusions.

In any case, a positive effect of NAC administration in combination with oseltamivir, an antiviral drug, has been demonstrated in a murine model of influenza infection [40].

There are other pharmacological effects of thiols that are also of potential relevance to the use of these drugs in patients with COVID-19. For example, NAC at 5 mM decreased the binding of ATII to its AT1R in vascular smooth muscle cells in a concentration-dependent model resulting in a decrease in ATII-stimulated signal transduction that was proportional to the decrease in receptor binding [41]. It should be noted, however, that the concentrations that decreased ATII binding (3–5 mM) in this experimental study did not differ from the plasma concentrations of NAC obtained after intravenous treatment of acetaminophen overdose. [42], whereas the plasma levels achieved after conventional oral dosing (1200 mg) are at least tenfold lower [43].

NAC, via GSH synthesis, was able to reverse the platelet activation, protein glycation, and pro-coagulation responses and protected against thrombosis, in the diabetic brain [44]. It also induced normalization of intraplatelet GSH, coupled with a reduction in platelet–monocyte conjugation, in patients with type 2 diabetes who were deficient in intracellular GSH [45].

In rats, erdosteine prolonged prothrombin time (PT) and activated partial thromboplastin time (aPTT), lowered the plasma levels of factors II, VII, VII, IX, and X, and reduced the antithrombin level [46]. However, at a dosage compatible with that used in humans, the platelet counts were not altered with erdosteine treatment [47], although in some experimental models, erdosteine decreased ischemia–reperfusion injury [48].

These pharmacological actions explain why NAC and thiol-based drugs in general are increasingly being proposed for the prevention and treatment of COVID-19, particularly ARDS associated with this disease [49–53].

## Evidence for Use of Thiols in COVID-19

**Fig. 1:**
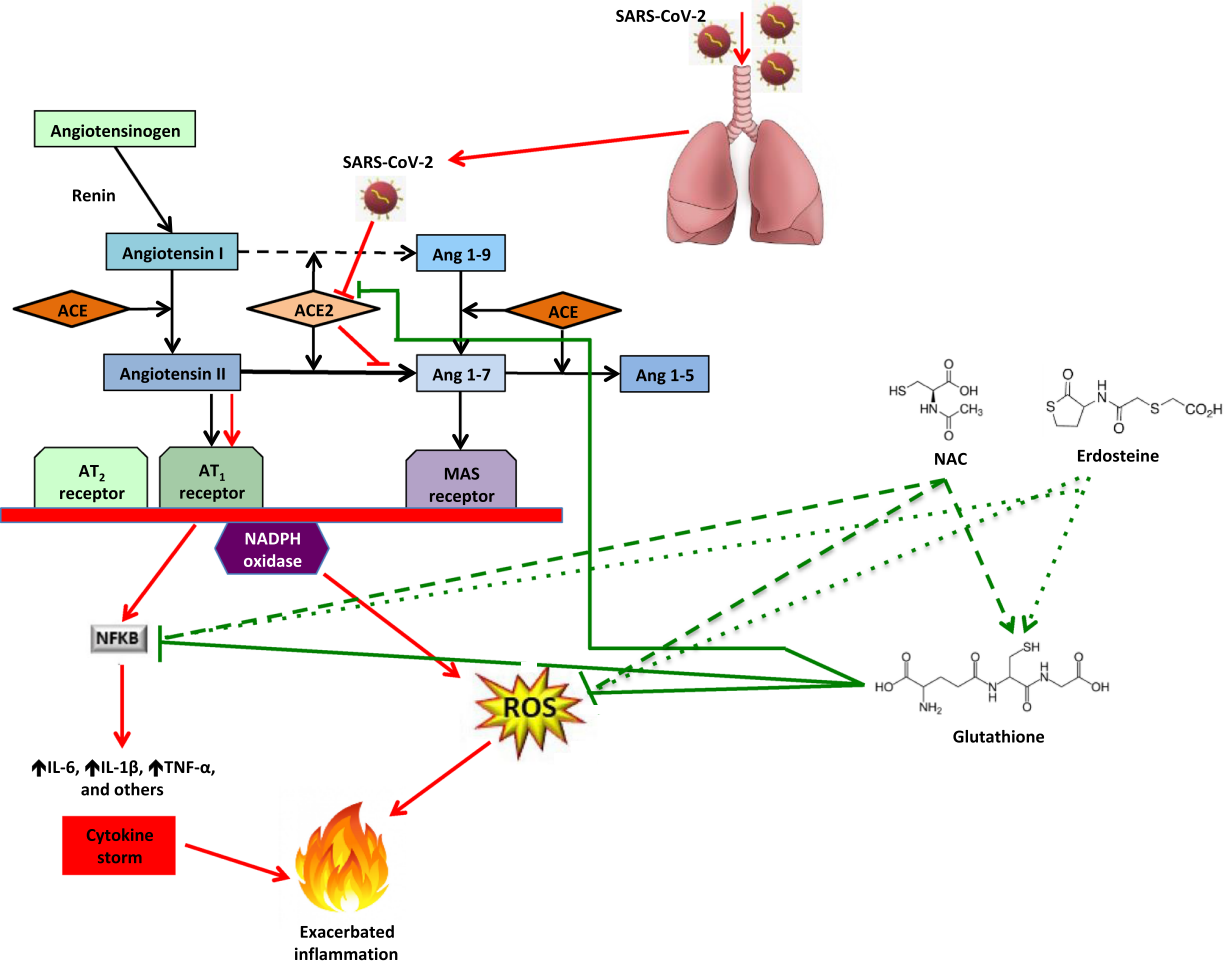
Possible mechanisms of action of thiols in the treatment of COVID-19

The importance of oxidative stress during COVID-19 and the different pharmacological properties of thiol-based drugs suggest a possible role for thiols in the treatment of COVID-19 (Fig. 1). Oral and intravenous GSH, as well as GSH precursors such as NAC, or drugs containing the thiol moiety (erdosteine) may represent a novel treatment approach for blocking NF-kB and addressing the “cytokine storm syndrome” and respiratory distress observed in patients suffering with COVID-19 pneumonia [54].

However, the reason for investigating thiols in the treatment of COVID-19 does not just rely on the evidence discussed above but should also take into consideration the effects these drugs can have on viruses.

The SARS-CoV-2 envelope (E) protein, which is involved in critical aspects of the viral life cycle, includes a triple Cys motif after the transmembrane domain that interacts with a similar motif from spike protein C terminus [55]. These two motifs could serve as a structural basis for the association between E and S, which would be mediated by the formation of disulphide bonds between the corresponding Cys residues. The association between E and S supports the hypothesis of an impact on virion stability by thiols [56]. It is plausible that NAC and erdosteine may cleave the two motifs that serve as a structural basis for the association between E and S; this would decrease SARS-CoV-2 infectivity [49]. However, thiols are likely to be ineffective against SARS-CoV-2 as a cell entry inhibitor as evidenced by the observation that even at NAC concentrations of 10 mM, it was impossible to observe a significant decrease in viral titre [57].

Nevertheless, thiols may still act against SARS-CoV-2 infection as they may exert their action through other diverse mechanisms. It has been hypothesized that thiols may attenuate methylglyoxal-induced protein glycation and further glycosylation events in SARS-CoV-2, which may then inhibit virus infectivity and associated pathologies [53]. Methylglyoxal and advanced glycation end products (AGEs) may play a role in the activation of inflammatory cells by binding to the receptor for AGEs (RAGE) and thus play a part in the pathogenesis of COVID-19 pneumonia and ARDS, as well as in lung inflammation.

However, it remained solidly held that the reduction of disulphides to sulphhydryl groups completely impairs the binding of the SARS-CoV-2 spike protein to ACE2 and provides a possible explanation for the molecular basis that affects the severity of COVID-19 infection due to oxidative stress [50].

In support of this hypothesis, it has been shown that NAC binds to Cys-145, the active site of the main protease that is required for viral replication, and thus inhibits the protease activity and viral replication [58]. Furthermore, proteomics data showed that NAC forms covalent conjugates with solvent-accessible Cys residues of spike protein that were disulphide bonded (Cys391-Cys525) in the native state [59]. In silico analysis indicated that this covalent conjugation perturbed the stereo-specific orientations of the interacting key residues of spike protein that resulted in threefold weakening in the binding affinity of spike protein with ACE2 receptor.

NAC is a precursor of Cys and this is the main substrate for hydrogen sulphide (H_2_S) production [60]. Several preclinical studies have provided evidence of antiviral activity of H_2_S [61]. Regarding more specifically COVID-19, H_2_S inhibits transmembrane protease serine 2 (TMPRSS2), a protease that promote ACE2 proteolytic cleavage using different targets in the protein sequence and amplifies SARS-CoV-2-entry via the endocytic pathway [48]. Furthermore, the ability of H_2_S to modulate the inflammatory response and proinflammatory cytokine cascade has been recognized in other settings [56]. H_2_S also contributes to the maintenance of elevated levels of GSH and normalizes the balance between oxidized and reduced GSH [glutathione disulphide (GSSG)/GSH ratio] that is increased by oxidative stress [61, 62], preventing endothelial NO synthase (eNOS) uncoupling, which leads this enzyme to produce O_2_^−^ instead of NO [57]. Furthermore, H_2_S increases the expression of ACE2 [62].

In addition, due to its ability to break disulphide bonds, NAC disrupts the platelet aggregation and breaks the bond between blood cells and clotting factor, maintaining the fluidity of the blood and oxygen flow in that area [11]. Thus, NAC could reduce the activation of the coagulation cascade characteristic of severe COVID-19 [11].

Despite these interesting pharmacological actions of thiols that could be potentially useful in the treatment of COVID-19, there are very few clinical studies of this drug class in patients with COVID-19 and the data are currently controversial. In this regard, it is worth mentioning a noteworthy experimental study by Bartolini et al. documenting that cells infected with SARS-CoV2 showed a reduced response to NAC compared to uninfected control cells, probably due to a reduced ability of the infected cell to support de novo biosynthesis of GSH [63].

Apart from reports of individual cases treated with NAC [64–66], there are some small but interesting studies in the literature to be mentioned. Thus, in 10 COVID-19 patients NAC induced beneficial effects, mainly a significant overall reduction in inflammatory markers (CRP and ferritin) [67]. However, when NAC administration was discontinued there was a rebound of inflammation in six patients. In another study that compared the effect of vitamin C, vitamin E, NAC, and melatonin plus pentoxifylline as adjuvant therapy in 110 COVID-19 patients with moderate to severe pneumonia, the simultaneous use of NAC (600 mg twice daily every 12 h) and pentoxifylline showed the best effect in patients with severe symptoms [68]. This suggests that the inflammatory state may benefit more from the combined use of two antioxidants.

A two-centre retrospective Greek cohort study that included 82 patients with COVID-19 pneumonia reported lower rates of progression to severe respiratory failure, need for mechanical ventilation, and death when patients received NAC 1200 mg/day [69].

In a Russian case–control study, 24 consecutive patients with confirmed SARS-CoV-2 infection and radiological findings compatible with severe COVID-19 pneumonia were treated with NAC at a daily dose of 1200–1800 mg intravenously and 22 patients were included in the control group [70]. NAC therapy provided a significant improvement in oxygenation parameters and reduction in CRP, National Early Warning Score (NEWS) 2 scale, and length of hospitalization.

However, in a Brazilian study in which 67 patients with severe COVID-19 were randomized to receive NAC 21 g (approximately 300 mg/kg) for 20 h and 68 dextrose 5%, there were no differences between the two groups in the primary outcome (need for invasive mechanical ventilation) or secondary outcomes (mortality, ICU admission, invasive mechanical ventilation time) [71].

Another study that enroled 92 patients with COVID-19-associated ARDS was unable to find difference in the 28-day mortality rate between the group treated with NAC at an intravenous dose of 40 mg/kg/day as an adjunct therapy (25.5%) and that treated with placebo (31.1%) [72].

The efficacy of erdosteine in patients with COVID-19 has also been examined. In one real-life observational study, 20 patients with COVID-19-related pneumonia received usual care plus erdosteine (300 mg twice daily) for 15 days after hospital discharge following local standard operating procedures [73]. Patients reported significant improvements in health-related quality of life (HRQoL) assessed by St George’s Respiratory Questionnaire and dyspnoea at rest and during daily activities assessed by the Modified Medical Research Council (mMRC) scale of dyspnoea during daily activity, the BORG scale for dyspnoea during exertion, and Visual Analogue Scale (VAS) for dyspnoea at rest.

Although the contrasting clinical evidence, the pharmacology of thiols would suggest that they should be considered as additional therapy in patients at risk of pneumonia and other complications following infection with SARs-CoV-2 and as an additional treatment to standard of care in patients discharged from hospital following COVID-19. Actually, thiols have now been added to local guidelines as treatments to be considered as part of standard of care for the treatment of patients with COVID-19 in a number of countries. In addition, there are several ongoing clinical studies investigating the use of thiols in COVID-19. In the NCT04374461 trial, patients with severe COVID-19 infections are receiving NAC IV 6 g/day in addition to supportive and/or COVID-19-directed treatments at the discretion of the treating physician. The number of patients who are successfully extubated and/or transferred out of critical care due to clinical improvement and that of patients who are discharged from the hospital due to clinical improvement are the primary outcome measures. The NCT04792021 trial is evaluating the effect of NAC 600 mg per os on oxidative stress and occurrence of complications in COVID-19 patients. The mean change in TNF-α is used to assess NAC efficacy. The large NCT04455243 trial that has planned to enrol 1180 participants aims to evaluate the efficacy of oral/IV NAC therapy (150 mg/kg every 12 h for 14 days) in the management of adult admitted patients with COVID-19. Time to recovery is the primary outcome measure. The purpose of the NCT04419025 trial, which has completed the recruitment, but no results have been posted to date, has been to evaluate the efficacy of oral NAC in preventing COVID-19 from progressing to severe disease. Furthermore, it has been reported that there are plans for additional studies to investigate the effect of erdosteine in COVID-19 prophylaxis [74].

Pending the results of these trials, in view of the scarcity of useful treatments against COVID-19 and the clinical rationale supporting the use of antioxidant drugs, an empirical therapeutic approach with thiols such as NAC and erdosteine should still be considered. Shi and Puyo [75] have suggested that the use of oral NAC (600 mg, bid) could be an effective and inexpensive measure to modulate the immune system against potential infection in those who have not contracted SARS-Cov-2, whereas when symptoms such as fever or dry cough appear, oral NAC (1200 mg, bid) could be taken to relieve symptoms and speed recovery from viral infection. Of course, the use of erdosteine orally at 600 mg or 900 mg may equally be recommended. Once patients develop clinically confirmed pneumonia or dyspnoea, in addition to regular therapy, such as remdesivir, IV NAC should be given intermittently or continuously. NAC can be infused at a dose of 100 mg/kg for at least 3 days. When a patient develops acute respiratory distress syndrome, along with regular antiviral therapy, 150 mg/kg at the first day, followed by a dose of 100 mg/kg/day for at least 3 days should be administrated to avoid irreversibly fatal multiple organ failure (MOF). However, once MOF or critical sepsis occurs, patients likely will not benefit from the administration of any thiol.

We feel it is our duty to highlight that these are only suggestions for those who want to treat their patients with COVID-19 also using a thiol-based drug. It is likely that data from the various ongoing trials and, perhaps, also the emergence of observational studies may lead to substantial changes in these suggested treatment regimens.
